# Behavioral Artistry: Examining the Relationship Between the Interpersonal Skills and Effective Practice Repertoires of Applied Behavior Analysis Practitioners

**DOI:** 10.1007/s10803-019-04082-1

**Published:** 2019-05-24

**Authors:** Kevin Callahan, Richard M. Foxx, Adam Swierczynski, Xing Aerts, Smita Mehta, Mary-Ellen McComb, Susan M. Nichols, Gabrielle Segal, Andrew Donald, Rachita Sharma

**Affiliations:** 1 Kristin Farmer Autism Center (KFAC), University of North Texas (UNT), 490 S. Interstate 35 East, Denton, TX 76205 USA; 2 Pennsylvania State University College of Medicine, Hershey, USA; 3 Department of Educational Psychology, UNT, Denton, USA; 4 Department of Rehabilitation and Health Services, UNT, Denton, USA

**Keywords:** Autism spectrum disorder, Applied behavior analysis, Social validity, Evidence-based practices, Therapeutic alliance, Behavioral artistry

## Abstract

This study investigated interpersonal skills associated with the concept of behavioral artistry (BA), a repertoire of practitioner behaviors including care, attentiveness, and creativity, among others, associated with the effective delivery of applied behavior analysis (ABA) treatment. Survey results indicated parents of children with autism preferred BA descriptors for ABA therapists over non-BA descriptors. A separate survey of 212 university students on a standardized personality assessment revealed students majoring and/or working in the field of ABA had lower levels of BA than those in other human services professions. Practitioners with higher BA scores were observed and rated more positively in their delivery of ABA for children with autism. Implications for training/supervising effective ABA practitioners within a BA model are discussed.

The evidence for the long-term effectiveness of applied behavior analysis (ABA) in the treatment of autism spectrum disorder (ASD) is vast (Foxx 2016). Individuals with ASD have been educated and treated using ABA for more than five decades (Ferster and DeMyer 1961, 1962; Lovaas et al. 1965; Wolf et al. 1964). Ten years ago, there were more than 1000 peer-reviewed scientific articles documenting ABA successes in autism (Foxx 2008), and that number has grown exponentially since (Volkmar 2015). Specific ABA interventions are considered evidence-based practices (EBPs) in autism (National Autism Center 2015; Peters-Scheffer et al. 2011; Schreibman et al. 2015; Smith 2001, 2013; Wong et al. 2015) and have been socially validated (Callahan et al. 2008, 2017).

ABA uses methods derived from scientifically established principles of behavior to improve the human condition (Baer et al. 1968; Cooper et al. 2007). It has been applied successfully to a range of populations and areas besides autism, including intellectual disabilities, all forms of education, business, mental health, counseling, medicine, and child abuse (Holdsambeck and Pennypacker 2016). Nevertheless, the incidence of ASD continues to increase and the majority of persons entering the field of ABA work with individuals with this disorder (Leaf et al. 2016). Over time, the conclusion that ABA is the treatment of choice (Schreibman 2005) and the gold standard of intervention for individuals with autism has become commonplace (Foxx 2016; Maurice et al. 1996, 2001; Mayville and Mulick 2011; Myers and Johnson 2007).

Despite the widespread acceptance of ABA as effective in the treatment and education of ASD, it has not escaped criticism (Schreibman 2005). Appraisals of ABA have suggested that its jargonistic language may be seen by others as abrasive, harsh, and unpleasant (Critchfield et al. 2017; Foxx 1996, 1998) and that ABA’s “drills and routines are cruel, and its aims misguided” (Devita-Raeburn 2016). Consumers often view the highly structured nature of some ABA treatment procedures as overly “mechanical,” and children’s responses to ABA’s Discrete Trial Training (DTT) protocols have been called robotic, prompt dependent, and lacking in both spontaneity and generalization of treatment effects (Schreibman 2005). While many of these and other critiques have been addressed and redressed (e.g., Foxx 2016; Maurice et al. 1996; Morris 2009), lingering related issues may be responsible for lower levels of social validity for the ABA approach when compared to other autism treatment models (Callahan et al. 2010). An important implication is that treatments having goals, methods, and/or outcomes that are not fully supported by their consumers may be implemented less frequently and/or less effectively (Smith 2013; Wolf 1978).

In a recent review of the skills needed for ABA practitioners to conduct effective programming for individuals with ASD, Leaf et al. (2016) identified intervention components related to what they describe as a “progressive” and “responsive” approach to the delivery of ABA services. These skills highlight interventionists’ abilities to be flexible and analytical in the implementation of individualized protocols and practices (for example, while using established EBPs such as DTT and functional analysis), rather than strictly adhering to today’s frequently observed “recipe-based” ABA approach (Leaf et al. 2016, p. 721). According to Leaf and colleagues, the pervasive use of ABA in autism treatment has resulted in changes in its scope and focus, and behavioral interventions have become potentially less effective:

A danger inherent in any large scale, quickly growing area is a loss of focus on meaningful purpose, process, and outcomes. In the field of ABA, this might translate into dogmatic lack of attention to clinical significance, selection of impractical procedures, ritualistic data-collection, over-abundant use of off-putting, dehumanizing terminology, disregard of logistical realities, and insensitivity to consumer issues (Leaf et al. 2016, p. 728).

To a large extent, Leaf et al. illuminate a critical need to assess the essential technical skills and related characteristics of the individuals delivering ABA services in order to ensure the future application of ABA as a highly effective and viable treatment approach for persons with ASD.

Eikeseth (2010) also investigated specific knowledge and skill components necessary to become a technically “competent” provider of early intensive behavioral interventions (EIBI) for children with autism, providing recommendations for assessing and training interventionist skills in the areas of basic intervention, comprehensive curriculum programming, working with families, and supervision. Eikeseth concluded that ineffective EIBI programming is related, in part, to deficiencies in meeting standards of overall program quality: “Highly intensive teaching and supervision will not produce optimal gains if teachers and/or supervisors do not have the necessary qualifications” (Eikeseth 2010, p. 243). Eikeseth’s conclusion naturally begs the question, what *are* the necessary qualifications of highly competent behavioral practitioners in autism?

In a discussion summarizing 25 years of lessons learned in the development and application of ABA practices, Foxx (1998) highlighted a concept from an earlier paper (Foxx 1985) wherein he concluded that the effectiveness of ABA may be negatively impacted by a broad array of deficits in the behavioral repertoires of practitioners. Similar to Leaf et al. (2016), Foxx asserted that there can be important differences in the outcomes achieved by interventionists delivering ABA services in a strictly traditional way (referred to by Foxx as “Behavioral Technologists” (BT)) and those who demonstrate important humanistic, interpersonal behaviors. He observed that some therapists, including individuals with little or no formal behavior analytic training, are qualitatively better at changing behaviors than their peers with equivalent or even greater levels of training. Foxx described these therapists as “Behavioral Artists” (Foxx 1998, p. 14). BTs, according to Foxx, often appear to be practitioners who simply learn a set of scripted, manualized strategies, methods, and procedures, and deliver them without much focus on the overall quality of the therapeutic interaction.

In contrast to technologists, Behavioral Artists are described as “natural behavior analysts” who demonstrate a set of seven interpersonal characteristics, as well as effective communication skills (Foxx 1998, p. 14). Foxx hypothesized that therapeutic skills associated with the concept of “behavioral artistry” (BA) include:

- Likes people: is able to establish rapport; demonstrates concern; wants to facilitate positive change;
- Has “perceptive sensitivity”: pays careful attention to important indicators of client behavior that may be small, subtle, and gradual;
- Doesn’t like to fail: sees difficult clients as a personal challenge to overcome, and as an opportunity for the client to succeed;
- Has a sense of humor: recognizes and accepts that much in the educational and human services professions is bizarre, illogical, and humorous;
- Looks “for the pony”: is optimistic and sees behavior change in a “glass half-full” context; always believes programming will be successful; is less likely to burn out;
- Is thick-skinned: doesn’t take negative client actions towards herself or himself personally; maintains objectivity and positivity; and
- Is “self-actualized”: does whatever is necessary and appropriate to facilitate and produce positive behavior change; is not under audience control; is creative (Foxx 1985, 1998).

Simply put, these therapeutic relationship skills are a foundational element of the concept of BA.

Several of Foxx’s BA components are similar to therapist skills in clinical psychology and counseling, and have long been associated with psychodynamic and humanistic/experiential approaches to behavior change. For example, the concept of therapeutic alliance (TA) refers to the collaborative, caring partnership that characterizes a positive client–therapist relationship (Lejuez et al. 2006). TA is seen as a fundamental component of effectiveness within other therapeutic approaches and has been demonstrated to be a significant predictor of treatment success (Holmqvist 2013; Horvath et al. 2011; Lejuez et al. 2006). Kerns et al. (2018) concluded that a strong TA was related to more positive treatment outcomes in high-functioning children with autism. Similarly, the related concepts of “empathic teaching” in special education (Morgan 1991) and “rapport building” (Shireman et al. 2016) and “compassionate care” (Taylor et al. 2018) in behavior analytic treatment have been highlighted in efficacy literature as essential repertoires among service providers for individuals with disabilities. The influence of empathy, in particular, has been frequently reported within medical and clinical care studies to be a key determinant of positive patient–client relationships and outcomes (e.g., Chaitoff et al. 2017; Derksen et al. 2013; Riess et al. 2012). Nevertheless, the roles and possible significance of these kinds of therapist behaviors in ABA have not been fully explored.

Further support for the Foxx and Leaf et al. conclusions about the need for a broader, more flexible approach in ABA treatment can be found in a 2013 article by Tristram Smith entitled, “What is Evidence-Based Behavior Analysis?”. In discussing the widespread use of treatment manuals, Smith noted concerns voiced by behavior analysts (Johnston et al. 2006) and professionals in other disciplines (Addis and Krasnow 2000) that strict adherence to manuals may stifle the delivery of services by expert providers. Smith posited that the “large and complex literature of…ABA procedures raises the possibility that there really could be an advantage to letting expert providers rely on their knowledge of the literature and their experience rather than a manual” (Smith 2013, p. 19). He cited Foxx’s description of behavioral artists having an uncanny knack for creating and administering effective ABA interventions, and suggested it might be worthwhile to evaluate whether providers are more effective without a manual. If so, he suggested that “the next step would be to conduct research that seeks to encapsulate what these providers do so others can replicate it” (Smith 2013, p. 19).

Given the observations by Leaf et al. (2016), Foxx (1985, 1998), and others suggesting that some persons delivering ABA services for individuals diagnosed with ASD lack essential prerequisite repertoires necessary to achieve optimal outcomes, it is important and potentially beneficial to investigate the qualities of ABA practitioners in autism. More specifically, if Foxx’s contention is true that ABA practitioners can lack some of the important interpersonal skills associated with BA, is it possible that some persons choosing to work in the field of ABA with the intention of providing direct services for individuals with autism possess a fundamentally different repertoire and/or therapeutic skills than those in other human services professions such as special education, speech and language pathology, and other fields? Further, could autism service providers who demonstrate the broader repertoires associated with BA be qualitatively more effective in the delivery of ABA practices than those without these repertoires? If so, would they attain more positive therapeutic outcomes? If the interpersonal components of BA can be validated as a preferred and advantageous skillset in autism treatment, and if behavioral artists can be reliably identified, there may be significant implications for screening, hiring, training, and supervising ABA practitioners who can best deliver evidence-based autism interventions. Finally, in keeping with Smith’s (2013) recommendations, it is also important to examine if autism service providers who do not initially display or possess comprehensive therapeutic repertoires can be effectively trained to demonstrate them with fidelity.

The purpose of this study was to investigate whether Foxx’s concept of BA could be validated and reliably measured using a standardized assessment, and to determine whether individuals studying and/or working in the field of ABA differ from those in other human services professions on important interpersonal skills potentially related to therapeutic effectiveness in autism treatment. In addition, we investigated the social validity of characteristics associated with the concept of BA with the parents of children with ASD. Finally, we assessed preliminary data to determine if there are differences in the quality of ABA treatment delivered by autism interventionists who have high or low levels of BA characteristics.

## Method

### Procedures and Instruments

#### Measuring Behavioral Artistry

Our research process required the identification of a reliable way to define and measure interpersonal characteristics associated with the repertoire of BA. The research team considered whether to develop an original assessment instrument or identify an existing standardized measure that accurately represented Foxx’s seven BA characteristics. After defining the Foxx BA traits and systematically reviewing several possible instruments, the Sixteen Personality Factor Fifth Edition Questionnaire (16PF homepage 2016; Cattell et al. 2013) was selected. The 16PF was developed by Cattell in 1949 and is currently in its fifth revision as a widely researched and used, comprehensive, self-report measure of normal adult personality (Institute for Personality and Ability Testing [IPAT] 2009). Designed for individuals aged 16 years and older, the 16PF has been implemented in a variety of research and applied settings (including clinical, counseling, and educational contexts) and has been used to determine and predict levels of creativity, leadership, interpersonal skills, and occupational profiles (Cousineau et al. 2007; Pietrzak et al. 2015). The validity and reliability of the 16PF instrument have been well established in more than 4000 research publications during the past 60 + years (IPAT 2009).

The fifth edition of the 16PF contains 185 multiple-choice items asking simple questions about the respondent’s daily behavior, interests, and opinions. Examples of questions include: “I’d enjoy more being a counselor than an architect (true; false),” and “I believe more in ___ (being properly serious in everyday life; the saying ‘laugh and be merry’ most of the time).” Each question has two narrative choices (“a” or “c”) as well as a “b” choice indicated by a question mark (“?”). Respondents are encouraged to choose the “b” (“?”) response only when neither of the other choices was a better descriptor, and are informed that there are no “right” or “wrong” answers.

The untimed, online version of the 16PF used in the present study takes approximately 30 min to complete. Responses result in scores on 16 primary personality factors along a bi-polar continuum (i.e., each personality factor is represented by two discrete poles, each having a unique, meaningful definition representing a different behavioral profile). For example, on the 16PF personality factor of “Warmth” respondents can score as being closer to the pole “Reserved, impersonal, distant” or the pole “Warm, outgoing, attentive to others” (IPAT 2009, p. 24).

For this study, the 16PF factor measuring “Reasoning” as a brief measure of intelligence was omitted from consideration. Previous research concluded that this factor is only a modest measure of intelligence (Abel and Brown 1998) as assessed by the 16PF items representing verbal, numerical, and logical reasoning. Although the 16PF test developers suggested that cognitive reasoning moderates the expression of personality traits, they recommended cautious interpretations of the Reasoning factor results. Thus, we questioned its relevance to the BA characteristics and the delivery of ABA treatment. For the remaining 15 personality factors, 38 raters located in two regions of the United States identified which factor poles were considered to be most closely associated with Foxx’s original conceptualization of BA. The raters included university faculty, doctoral students in special education and behavior analysis, and master’s level practitioners who delivered direct services to individuals with ASD. Poles for three personality factors—Warmth, Liveliness, and Perfectionism—had 100% agreement as being related to BA, while poles for the other 12 factors were determined to be associated with BA with strong agreement across raters. During this process, both poles for several of the factors were determined to be relevant components of BA. These personality factors were referred to as “Cusps” by the research team. Table 1 lists the 15 personality factors and their poles determined to be BA-compatible and non BA-compatible, as well as those factors considered to be Cusps.

**Table 1:** 16PF primary factors, behavioral artistry poles, non-behavioral artistry poles, and cusps

16 PF primary factor	Behavioral artistry pole	Non-behavioral artistry pole	Cusp? (both poles BA-compatible)
Warmth	Warm, outgoing, attentive to others	Reserved, impersonal, distant	No
Emotional stability	Emotionally stable, adaptive, mature	Reactive, emotionally changeable	No
Dominance	Dominant, forceful, assertive	Deferential, cooperative, avoids conflict	No
Liveliness	Lively, animated, spontaneous	Serious, restrained, careful	No
Rule-consciousness	Rule-conscious, dutiful	Expedient, nonconforming	Yes
Social boldness	Socially bold, venturesome, thick-skinned	Shy, threat-sensitive, timid	No
Sensitivity	Utilitarian, objective, unsentimental	Sensitive, aesthetic, sentimental	Yes
Vigilance	Trusting, unsuspecting, accepting	Vigilant, suspicious, skeptical, wary	No
Abstractedness	Grounded, practical, solution-oriented	Abstracted, imaginative, idea-oriented	Yes
Privateness	Forthright, genuine, artless	Private, discreet, non-disclosing	No
Apprehension	Self-assured, unworried, complacent	Apprehensive, self-doubting, worried	No
Openness to change	Open to change, experimenting	Traditional, attached to the familiar	No
Self-reliance	Self-reliant, solitary, individualistic	Group-oriented, affiliative	Yes
Perfectionism	Perfectionistic, organized, self-disciplined	Tolerates disorder, unexacting, flexible	No
Tension	Relaxed, placid, patient	Tense, high energy, impatient, driven	No

### Factor Examination, Model Comparison, and Model Modification

Statistical modeling was conducted to further analyze the 15 personality factors in order to determine which factors had the greatest explanatory and predictive power related to the concept of BA. A parsimonious model is the simplest model with the least assumptions and variables, while also having the desired level of explanation and prediction. Factor examination, model comparison, and model modification are typically used to analyze each factor in a statistical model to ensure its effectiveness in explaining observed results (Judd et al. 2009). In this case, after each of the 16PF personality factors was examined, taking into consideration their individual meanings, relationship with the Foxx traits, individual factor analysis of variance (ANOVA) results, mean plot shapes, and commonalities from the factor analysis results, statistical modeling identified only eight of the 15 personality factors as clearly supportive of the concept of BA. During these analyses, most of the Cusp poles were dropped from the BA factor combination because they did not fully support the overall BA percentage score as a main factor. Using these statistical modeling results, all of Foxx’s BA characteristics were determined to be directly supported by one or more of the 16PF personality factors retained in the model.

In conclusion, as a result of our statistical modeling, a behavioral artist was hypothesized to represent interpersonal and therapeutic behaviors associated with warmth, emotional stability, liveliness, social boldness, self-assurance, openness to change, self-reliance, and perfectionism (see Table 2).

**Table 2:** Final 16PF poles associated with behavioral artistry with parent preference percentages, and corresponding Foxx behavioral artistry characteristics

16PF pole associated with behavioral artistry (percentage of parent preference)	Foxx (1998) behavioral artistry characteristics
Warm, outgoing, attentive to others (82.6%)	Likes people
Emotionally stable, adaptive, mature (94.2%)	Thick-skinned
Lively, animated, spontaneous (82.6%)	Likes people; looks for the pony
Socially bold, venturesome, thick-skinned (96.5%)	Self-actualized; thick-skinned
Self-assured, unworried, complacent (75.6%)	Self-actualized
Open to change, experimenting (93.0%)	Perceptive sensitivity; sense of humor
Self-reliant, solitary, individualistic (74.4%)	Self-actualized
Perfectionistic, organized, self-disciplined (94.2%)	Does not like to fail

#### Parent Validation

A national sample of parents of children with ASD (*n *= 86) completed an online survey designed to assess their opinions about the personality traits comprising the concept of BA. For each personality factor, parents were asked to select their preferred description of interpersonal behaviors associated with either the 16PF pole determined to be compatible with BA or the non BA-compatible pole (see Table 1). Specifically, respondents were instructed to indicate which of the descriptions they believed was more desirable for service providers in autism. The survey questions intentionally did not use the names of the factors or pole descriptors in order to prevent biasing the respondents. For example, the survey question addressing the factor of “Warmth” stated, “Factor A measures a person’s emotional orientation toward others—the degree to which contact with others is sought and found to be rewarding as an end in itself. People who score A + generally like and need to be around other people. They rarely like to be alone. They need and want high levels of interaction with other people. People who score A − generally like to interact with ideas more than people. They may like and value other people but tend not to find social interactions rewarding. They enjoy solitary activities.” For all personality factors, parents selected a choice indicating whether they would rather have people who scored “+” or “−” work with their child with autism. The BA-compatible poles included both “+” and “−” descriptors.

#### 16PF Survey

In order to determine if there was a difference in the level of BA characteristics among university students majoring in ABA and other major areas of study, we conducted an online survey using the 16PF. Undergraduate and graduate students in the majors of ABA, special education, rehabilitation counseling, and other human services majors completed the survey. “Other human services” majors included students in speech and hearing sciences, clinical counseling, psychology, child development, occupational therapy, adapted physical education, and similar majors. Additionally, engineering and computer science students served as a comparison group of persons not expected to pursue professional careers working in human services with individuals with autism or intellectual disabilities.

The 16PF survey results for each respondent were analyzed. For each personality factor the respondent’s scores indicated whether he or she fell into the BA-compatible pole or the non BA-compatible pole category. Overall percentages of BA characteristics were then computed for each respondent. Mean percentages were computed for three main survey respondent groups: (1) combined group (all respondents, including undergraduate and graduate students working at the autism center and students external to the autism center); (2) autism center group (respondents who were employed part-time or full-time at the university-based autism center delivering ABA therapy); and (3) external group (student respondents not working at the autism center).

In order to compare the BA characteristics of participants across majors and groups, we conducted one-way Analyses of Variance (ANOVA) on individual 16PF personality factors, as well as overall BA percentage scores. Bonferroni post hoc analysis was examined for each of the ANOVAs.

#### Therapist Observations

To determine if autism interventionists with higher and lower levels of BA characteristics deliver ABA services differently, observations were conducted of behavioral therapists at a university-based autism treatment center who completed the 16PF survey and scored in the highest and lowest quartile among participants. The survey scores of therapists in the High-BA percentage group indicated these service providers had six or more of the eight BA characteristics, while those in the Low-BA group had two or fewer of the BA characteristics. Ten-minute periods of DTT and naturalistic environment training (NET) therapy sessions were videotaped using a handheld video camera with a remote microphone placed near the point of therapy delivery. All therapists were videotaped a total of six times. The initial three videotapes were designed to limit reactivity by the therapists and clients to the presence of the data collector. Only the final three videotaped therapy sessions were scored for BA. The sessions scored occurred on different days and times, and sometimes with different clients. Data collectors were blind to the BA category of the therapists (i.e., High-BA or Low-BA).

Pair-wise comparisons were conducted using the *t* test for independent samples between therapists considered to be High-BA and Low-BA across four qualities of the Foxx trait Likes People, subjective ratings of the overall quality of therapy, and therapist levels of behavioral technology. T-tests for independent samples comparisons were also made between High-BA and Low-BA therapists across these same factors during the delivery of DDT and NET therapy sessions.

#### Behavioral Artistry/Likes People

The research team posited that therapist behaviors associated with the Foxx BA characteristics “Likes People,” “Thick-Skinned,” “Perceptive Sensitivity,” and “Sense of Humor” could be observed during the delivery of typical DTT and NET programming. Operational definitions for each of these BA characteristics were developed. However, during field testing it became apparent that only behaviors associated with “Likes People” occurred frequently enough during therapy sessions to measure meaningfully as a component of BA. It was further observed that behaviors associated with the Foxx characteristics of Thick-Skinned and Sense of Humor were subsumed within the definition of “Likes People.”

“Likes People” was generally defined as observable demonstrations of enjoyment and concern directed towards a client, with four associated behavioral indicators: (a) pleasant facial expression; (b) positive tone of voice; (c) sustained gaze at the client; and (d) body proximity and orientation toward the client. “Likes People” could only be scored during times the therapist was engaged in social interactions with clear communicative and therapeutic intent. The occurrence or non-occurrence of the four indicators of “Likes People” was scored by data collectors using a partial interval data sheet, on which a 10-min observation period was divided into 10-s scoring intervals. If the behavioral indicator was observed at any time during an interval, data collectors marked a “+” on the data sheet. A total percentage of occurrence was computed for each of the four behavioral indicators for each observation session. Data collectors were required to demonstrate mastery of the scoring system before beginning BA scoring.

In addition to partial interval scoring of the behavioral indicators of “Likes People,” data collectors recorded a subjective rating of the therapist’s behavior throughout the entire 10-min therapy session, based on a standardized description of typical examples of the target behavior, as follows: “Likes People will typically appear as a person who is fun, friendly, and childlike; energetic, positive, and affectively expressive; uses appropriate physical touch and gestures; appears attentively interested in what the client is doing; is engaged in activities that demonstrate care for the client’s welfare and happiness; demonstrates empathy, respect, and politeness”. At the conclusion of a scoring session, data collectors subjectively rated the therapist’s “Likes People” behaviors on a scale of zero to one hundred. A rating of 100 was defined as the therapist demonstrating 100% of the above criteria continuously and at the highest possible levels throughout the entire session. A rating of zero indicated that none of the subjective indicators were present at any time, in any way, during the observation session. All data collectors demonstrated mastery levels of agreement with a scoring key in making subjective ratings of therapist behaviors before beginning independent data collection.

#### Behavioral Technology

The videotaped therapy sessions were used to separately rate the quality of BT skills, defined as the fidelity of implementation of previously trained DTT and NET therapy components. For example, therapists delivering DTT programs were rated using an event recording system on the occurrence or non-occurrence of eight specific skills related to gaining the client’s attention, appropriate presentation of stimuli, correctness of prompting, and delivery of reinforcement, among others (Leaf and McEachin 1999). An overall percentage of BT skills was recorded for each therapist for each of the three scored therapy sessions.

#### Interobserver Agreement (IOA)

Interobserver agreement was computed for 33% of the observation sessions using the scored interval method. The overall combined IOA was 94.2%. IOA for the individual components of Likes People were: pleasant facial expression = 88.2%, positive tone of voice = 90.4%; sustained gaze = 99.0%; body position and orientation = 99.1%.

### Participants

The student participants were recruited from a university in the Southwestern United States where 53% of the students were female, 47% male, 48% Caucasian, 22% Hispanic, 14% African-American, 7% Asian/Pacific Islanders, 2% Native American, and 1% identified as “Other” (six percent chose not to disclose). All student participants working at the university-based autism center had completed supervised training qualifying them for national certification as a Registered Behavior Technician (RBT). Autism center participants had worked at the center an average of 10.9 months (range 1.3–40.0 months). The parent participants recruited for the social validation portion of the study identified as a parent of a child with autism who was receiving services at an autism center located in Arizona, California, Colorado, Hawaii, Texas, Utah, or Washington.

## Results

### Parent Validation

Eighty-six parents of children with autism completed an online survey asking them to choose from two descriptors of 16PF characteristics representing BA or its corresponding non-BA pole. For each of the personality factors, parents preferred the BA-compatible trait descriptor (see Table 2). The mean percentage of parent preference for the BA characteristics was 86.6% (range 74.4 to 96.5%). These findings indicate that these parents overwhelmingly preferred the descriptions of therapist behaviors associated with the concept of BA when compared to interpersonal behaviors not associated with BA.

### 16PF Survey

A total of 212 students (134 undergraduates, 78 graduates; 170 female, 42 male) completed the online version of the 16PF. One hundred eight of the respondents worked part-time or full-time at a university-based autism center delivering ABA therapy. Table 3 details the number of individuals within each major area of study. Results were analyzed using ANOVA and other statistical analyses comparing groups by overall percentage of BA characteristics or by raw scores on individual personality traits. Additional analyses were conducted using the t-test for independent samples, comparing groups consisting of the highest scoring (High-BA) and lowest scoring (Low-BA) respondents.

**Table 3:** Number of 16PF survey completers by student status and major area of study

Major area of study	Number of 16 PF survey completers (*n *= 212)				
	Overall total	Undergraduate students (*n* = 134)		Graduate students (*n* = 78)	
		Autism center (*n* = 92	External (*n* = 42)	Autism center (*n* = 16)	External (*n* = 62)
Applied behavior analysis	49	29	4	6	10
Special education	31	9	0	4	18
Rehabilitation counseling	35	3	20	1	11
Other human services	61	43	2	4	12
Non-human services	36	8	16	1	11

### ABA Compared to Other Major Areas of Study

Table 4 shows the mean percentages of BA characteristics for all groups, including Combined, Autism Center, and External, across major areas of study. Students majoring in ABA had the lowest overall levels of BA characteristics across all human services majors. There were no statistically significant differences between ABA majors and other majors. In the combined group, a statistically significant difference was observed between Special Education majors and Non-Human Services majors, F(4207) = 3.47, *p *= 0.009. In the external group, a statistically significant difference was also observed between Special Education and Non-Human Services majors, F(4,99) = 2.84, *p *= 0.028. As expected, respondents in the Non-Human Services comparison group (i.e., students majoring in engineering or computer sciences) had the lowest overall mean percentages of BA characteristics within both the Combined and External groups. The results within the Autism Center group were surprising in that practitioners who were ABA majors had a lower mean percentage of BA characteristics than respondents within *all* other major areas of study, including the Non-Human Services majors.

**Table 4:** Mean percentage of behavioral artistry characteristics by major area of study

Survey respondent group	Mean percentage of behavioral artistry characteristics				
	Applied behavior analysis	Special education	Rehabilitation counseling	Other human services	Non-human services
Combined	42.6	54.4*	45.4	49.4	39.6*
External	40.2	53.5**	44.4	45.5	34.7**
Autism center	43.6	55.8	53.1	50.5	54.2

*Statistically significant difference for combined group data: F(4, 207) = 3.47, *p* = 0.009. Bonferroni post hoc analysis indicates statistically significant difference between special education and non-human services majors (*p* = 0.015)

**Statistically significant difference for external group data: F(4, 99) = 2.84, *p *= 0.028. Bonferroni post hoc analysis indicates statistically significant difference between special education and non-human services majors (*p* = 0.016)

Table 5 details the results of analyses of the 16PF individual personality factors. Our pair-wise comparisons of the eight factors identified two statistically significant differences between ABA students and respondents in other academic majors. On the personality factor of “Warmth,” ABA majors in the External Group had the lowest overall mean scores of all groups, including Non-Human Services majors. This difference was a statistically significantly lower level of warmth than respondents in the Rehabilitation Counseling group, *p *< 0.000. Unexpectedly, ABA majors in the External Group also had the lowest overall mean scores of all groups on the factor of “Perfectionism,” statistically significantly lower than Special Education respondents, *p *= 0.031.

**Table 5:** Mean raw scores on individual 16PF factors by major area of study

16PF factor (survey respondent group)	Mean raw scores				
	Applied behavior analysis	Special education	Rehabilitation counseling	Other human services	Non-human services
Warmth (external)	4.14*	5.39	6.16*	5.93*	4.19*
Perfectionism (external)	4.57**	6.39**	5.45	5.14	5.59

*Statistically significant difference for External Group data on Warmth: F(4, 99) = 6.54, *p* < 0.000. Bonferroni post hoc analysis indicates statistically significant differences between applied behavior analysis and rehabilitation counseling majors (*p* = 0.005); rehabilitation counseling and non-human services majors (*p* < 0.000); other human services and non-human services majors (*p* = 0.032)

**Statistically significant difference for external group data on perfectionism: F(4, 99) = 2.51, *p* = 0.046. Bonferroni post hoc analysis indicates a statistically significant difference between applied behavior analysis and special education majors (*p* = 0.031)

### High-BA Group

Survey scores for respondents in the High-BA group (*n *= 13) indicated they had either six (75%) or seven (87.5%) of the BA characteristics. None had all eight (100%). Only one of the 13 (7.7%) was an ABA major (overall, 32.4% of Autism Center respondents were ABA majors). The personality factors most frequently occurring were Open to Change and Perfectionistic, with 92.3% of the High-BA therapists having these characteristics, and 84.6% having Warmth, Liveliness, Social Boldness, and Self-Assurance. The least frequently occurring personality factor was Self-Reliance (38.5%). Two of the 13 therapists (15.4%) were males (overall, 19.8% of the respondents were male).

### Low-BA Group

All respondents in the Low-BA Group (*n *= 13) had only two (25%) of the BA characteristics. Four respondents (30.8%) were ABA majors. The most frequently occurring personality factors were Self-Reliance (76.9%) and Perfectionism (69.2%). Only two of the 13 (15.4%) had the traits of Warmth or Liveliness. No therapist in the Low-BA group had the personality factors of Emotional Stability, Social Boldness, or Self-Assurance. As in the High BA group, there were two males (15.4%) in the Low-BA group.

### Therapist Observations

#### Behavioral Artistry/Likes People

Three videotaped recordings of 10-min ABA therapy sessions were rated for each therapist/respondent in the High-BA and Low-BA groups. Using partial interval scoring of 10-s intervals, a percentage of occurrence was scored for each of the components of the Foxx trait “Likes People,” based on operational definitions of Pleasant Facial Expression, Positive Tone of Voice, Sustained Gaze, and Body Proximity/Orientation. A combined mean level of occurrence across these four components was also computed.

Table 6 shows that therapists in the High-BA group demonstrated higher percentages of Behavioral Artistry/Likes People on all behavioral components, with the exception of Body Position/Orientation, for which scores were almost the same. Therapists in the High-BA group were rated to have a statistically significantly higher level of Pleasant Facial Expression than therapists in the Low-BA group, t(18) = 2.22, *p* = 0.040. All other comparisons were non-significant. A subjective rating (zero to 100) was also scored for each therapy session based on a standardized description of “Likes People.” Although the subjective ratings of therapists in the High-BA group were higher than those in the Low-BA group, this difference was not statistically significant.

**Table 6:** Mean ratings of therapist behavioral artistry/likes people and behavioral technologist observations

	Behavioral artistry/likes people						Behavioral technology
	PFE	PToV	SG	BPO	Overall mean	Subjective rating	
High-BA	31.27*	91.87	100.00	99.72	80.46	78.50	96.67
Low-BA	15.83*	89.49	97.58	99.90	74.94	71.88	94.00

*PFE* pleasant facial expression, *PToV* positive tone of voice, *SG* sustained gaze, *BPO* body position and orientation

*Statistically significant difference between High-BA and Low-BA groups for pleasant facial expression: t(18) = 2.22, *p* = 0.040

#### Behavioral Technology

Using the same videotaped sessions, separate ratings were made of each therapist’s behavioral technologist (BT) skills, defined as the percentage of steps completed in the implementation of DTT and NET therapies as prescribed at the autism center. The higher BT scores observed among therapists in the High-BA group compared to therapists in the Low-BA group (see Table 6) were non-significant.

In order to identify possible confounding variables, the gender and experience levels of the therapists were analyzed (see Table 7). Recall that there were an equal number of males in both groups. Females scored higher than males in both the High-BA and Low-BA groups. Therapists in the High-BA group averaged 7.9 months of experience working at the autism center, while the mean number of months of experience in the Low-BA group was almost 14 months. These findings suggest that the higher ratings in the High-BA group were not because of a gender disparity or because of greater levels of experience and related training at the autism center.

**Table 7:** Autism center therapist gender and experience levels by High-BA and Low-BA groups

	Therapist gender		Therapist experience at the autism center	
	Number of females (mean BA percentage)	Number of males (mean BA percentage)	Mean number of months	Range (months)
High-BA group	11 (81.89)	2 (74.28)	7.91	1.3–17.5
Low-BA group	11 (75.77)	2 (70.38)	13.98	4.0–40.1

The number of DTT and NET sessions within each group was also examined. As expected, BA/Likes People ratings for NET sessions were higher overall and on the subjective ratings. The High-BA group had a greater number of NET sessions than the Low-BA group (see Table 8). Within the DTT sessions there was a statistically significant higher level of Pleasant Facial Expression by the High-BA group, t(37) = 2.80, *p *= 0.008. No other comparisons were statistically significant. There was an interaction effect for the Low-BA group, which demonstrated a slightly higher level of Positive Tone of Voice than the High-BA group during DTT sessions, but a lower level of Positive Tone of Voice during NET sessions.

**Table 8:** Comparison of DTT and NET sessions by High-BA and Low-BA groups

	Total # sessions	Mean BA ratings (% of intervals): discrete trial training (DTT)					Total # sessions	Mean BA ratings (% of intervals): naturalistic environment training (NET)				
		PFE	PToV	SG	BPO	Subjective rating		PFE	PToV	SG	BPO	Subjective rating
High-BA	25	28.3*	87.8	100	99.6	75.2	14	34.4	99.2	100	100	81.4
Low-BA	34	14.1*	89.9	99.9	99.9	71.2	5	27.7	80.0	100	99.4	73.8

*PFE* pleasant facial expression, *PToV* positive tone of voice, *SG* sustained gaze, *BPO* body position and orientation

*Statistically significant difference between High-BA and Low-BA groups on pleasant facial expression for DTT: t(37) = 2.80, *p* = 0.008

## Discussion

At its core, this research attempts to answer the overarching question posed more than three decades ago by Richard Foxx: What are the behaviors that make some behavior analysts *better* than others? (Foxx 1985). In the development of his concept of Behavioral Artistry, Foxx hypothesized, like others more recently (e.g., Geller 2014; Leaf et al. 2016), that the repertoires of today’s behavior analysts can lack essential interpersonal behaviors related to the optimal delivery and outcome of ABA services. More pointedly, Taylor and colleagues concluded that behavior analysts “do not always establish or sustain collaborative and caring relationships” (Taylor et al. 2018, p. 2), and these skill deficits can have potentially negative impacts on treatment delivery and client outcomes.

Many of the behaviors corresponding with Behavioral Artistry, including, for example, being warm and perceptively sensitive to others, relate to a focus of behavioral research that once saw considerable attention (e.g., Cooke and Cooke 1974; Frostig and Maslow 1973; Smith 1974; Wandersman et al. 1976). However, the importance of establishing and maintaining caring interpersonal relationships between behavioral therapists and their clients has been virtually neglected in the more recent ABA treatment literature.

Foxx’s vision of a better behavior analyst resulted from his decades of both formal and anecdotal observations of persons providing ABA treatment in socially significant environments. He concluded that one can clearly recognize the difference between behavioral artists and BTs. Behavioral artists smile and laugh more. They pay attention and listen more carefully. They are, at the same time, more objective *and* more creative in the delivery of ABA therapy because they are self-aware and driven to produce positive outcomes for their students and clients. They are perpetually optimistic.

Our results, although preliminary and limited, largely support this view. Importantly, we confirmed that therapists with a larger percentage of BA characteristics look qualitatively better delivering ABA therapy for children with autism than those with fewer BA traits, at least on one key indicator of warmth, attentiveness, and liveliness—Liking People. In this case, “better” meant that behavioral artists simply looked like they enjoyed their clients more, and appeared to be more caring, positive, and pleasant delivering ABA treatment than therapists who were less behaviorally artistic. Nevertheless, additional replications of our results demonstrating statistically significant findings are necessary to support robust claims about their potential practical and clinical significance.

As in all preliminary research, important additional questions have emerged from this study. In this case, at least four key questions should be addressed with further research: (1) What are the relationships between the technology and artistry of ABA treatment in autism? (2) Do the behaviors/repertories associated with BA improve student/client outcomes? (3) Can the repertoires of BA be effectively trained? (4) What can the fields of ABA, special education and autism do to identify, recruit, and retain practitioners with positive interpersonal behaviors?

For the past several decades, the literature on ABA programming for children with autism has focused almost exclusively on improving the *technology* of treatment. Researchers and practitioners have done an exemplary job identifying EBPs and developing curricula to increase effective autism programming. Indeed, Foxx’s seminal articles which introduced the concept of BA (Foxx 1985, 1998) also included robust emphases on the necessary technological knowledge and skills therapists must possess in order to be effective providers of ABA treatments. In our study, therapists with both the highest and lowest levels of BA demonstrated relatively similar levels of technical competence. Thus, we can conclude that most ABA practitioners, if adequately trained and supervised, can deliver ABA protocols with technological fidelity. However, we believe the repertoires of BTs, although necessary, are not sufficient if we wish to continue to advance the development of the fields of behavior analysis and autism treatment in order to attain maximum, broad reaching clinical and educational impacts. We agree with recent calls for examining the qualities and corresponding behaviors of exemplary behavior analysts, including identifying how components of humanistic therapeutic care may be integrated within the delivery of high quality ABA treatment. As Taylor et al. (2018) point out, the empirically derived *technical skills* of behavior analysts will always remain a critically important component of client outcomes. Nevertheless, “those methods do not exist separately from relationships with clients and their caregivers” (Taylor et al. 2018, p. 1). Future research should investigate these relationships, and the potential synergies that could result from maximizing technical competence *in coordination with* BA repertoires. Such research could prove to be a valuable addition to the literature on the relationship between therapist interpersonal skills and effective practice (e.g., Anderson et al. 2009; Keijsers et al. 2000; Lambert and Barley 2001).

Formally investigating the relevant question of whether clients of behavioral artists attain more positive therapeutic outcomes was beyond the scope of this study. Hypothetically and logically, however, ABA therapists who are warm, attentive, creative, optimistic, and persevering should engage clients instructionally at higher levels and minimize escape-avoidance and problem behaviors, allowing for the more effective delivery of their corresponding technologist repertoires. Nevertheless, future research should examine the interpersonal characteristics and behaviors of ABA practitioners, with the intention of identifying more precisely the repertoires and behaviors associated with the most positive client outcomes. The social validation of these outcomes is vitally important (McMahon and Forehand 1983), especially for a field which continues to be subject to negative public perceptions and misperceptions, and less than universal acceptability of its methods and language (Critchfield et al. 2017; Foxx 1996; Woolfolk et al. 1977). Our confirmatory parent survey results suggest that at least this important consumer group supports the inclusion of the interpersonal aspects of BA as a future hallmark of ABA therapy in autism intervention.

Another focus for future research is to determine if autism practitioners with lower levels of BA can be taught to consistently demonstrate associated behaviors at a higher level. In addition to recent efforts within the field of ABA (e.g., Lugo et al. 2017; Shireman et al. 2016) other helping professions, such as counseling, have also concluded that the basic skills of establishing therapeutic rapport are, indeed, trainable (Carkhuff 2009; Ivey et al. 2018). We believe that by using ABA-based training methods such as Behavioral Skills Training (e.g., Parsons et al. 2012) therapists entering the field can be taught to recognize skill deficits and receive effective training to remediate them. Crucial questions will include how much improvement can be achieved and whether or not the observed changes are significant and appear genuine.

Finally, we believe that expanding the definition of effective ABA practitioners to include the characteristics of BA can ultimately improve the delivery, outcomes, and acceptability of ABA treatment for individuals with ASD. Unfortunately, our preliminary results suggest that persons with the highest levels of BA may often seek other human services professions than ABA in which to apply their therapeutic skills. It is unclear why the students in our study majoring in ABA had the lowest levels of BA, and, more concerning, the lowest levels of warmth, across all groups of human services providers. But this is a strikingly compelling finding. It could be beneficial for the field of ABA to conduct a large-scale self-study effort to investigate this phenomenon.

### Research Limitations

Several limitations need to be acknowledged as a function of the preliminary nature of this study. As most researchers would appreciate, any innovative concept is more likely to be refined through systematic replications over time. Limitations of this research include the relatively small sample size of therapists observed and the fact that we conducted observations within only one setting. Future replications should be conducted in a variety of environments including homes, schools, and clinics outside of university-based environments. Although this was addressed somewhat in the parent surveys, most of this study occurred at a university in the Southwestern part of the United States. It would have been ideal to conduct this research as a multi-site study to determine, for example, if there are any regional, cultural, or national differences in BA repertoires or parental expectations. Because the university autism center where this study was conducted already screens and hires therapists who demonstrate a certain level of BA, we would anticipate greater ranges of interpersonal behaviors within other settings.

A related possible limitation was that the observers in this study were former therapists at the autism center. Although they were blind to the category of the therapists they were observing, ratings could have been impacted by the data collectors’ personal knowledge of clients and staff. Additionally, a limitation was that we were unable to control for the number of NET versus DTT sessions because our random assignment of observed therapy sessions, which led to an over-representation of NET sessions. This disparity in sessions represented a possible confounding factor for our obtained results.

Another limitation is related to classification of the student respondents of the 16PF survey. Although we categorized respondents by majors, our analyses did not separately account for respondents who had different graduate and undergraduate degree majors (e.g., special education as an undergrad and ABA as a graduate student) or for respondents who may have had ABA certifications in addition to a graduate degree in a major other than behavior analysis. In the former case, we categorized respondents into the major of their most advanced degree. However, we did not account for the latter situation. It is possible that various combinations of education and training could have impacted our results.

Another limitation was our inability to investigate the Foxx BA traits of “Doesn’t like to fail” and “Looks for the pony,” as well as the communication skills of therapists. Future research should seek to conduct a component analysis of the full package of BA traits. Efforts should also be made to investigate the concept of artistry within different formats of ABA therapy in order to determine if any differences exist.

A final limitation is that we had a non-random sample. While it is not uncommon for such sampling methods in online survey research, they do present some limitations. Results cannot necessarily be assumed to represent the larger population. There is also the potential for bias based on individuals who self-selected to participate compared to those who elected not to participate.

## Conclusions

With its resolute emphasis on the primacy of socially relevant outcomes within the practice of ABA, Baer et al.’s seminal (1968) article is entrenched among a select number of publications which help guide an entire discipline’s conduct (Critchfield and Reed 2017). Yet, during the 50 years since its publication, the tendency of ABA to focus so exclusively on the prescribed application of *only* seven dimensions of practice may have unintentionally restricted the broader growth of the field. Critchfield and Reed (2017) report that even before Foxx raised the issue (Foxx 1985, 1998), researchers and practitioners questioned whether ABA had become “overly technological” (p. 12). Todd Risley himself reminded practitioners that he and Montrose Wolf always “assumed that the enterprise of Applied Behavior Analysis would evolve” (Risley 2001, p. 270) and that new opportunities in the development of ABA would undoubtedly arise (Critchfield and Reed 2017).

In presenting their “current dimensions of Applied Behavior Analysis” Baer and colleagues defined generality as “practical improvement in important behaviors” (Baer et al. 1968, p. 96). In autism treatment, what could be more important than improving specific behaviors of the practitioners applying ABA, in order to ensure the most socially valid and generalizable outcomes? These behaviors necessarily *must* include the ways in which behavior analysts establish and maintain positive, socially valid therapeutic relationships with their clients and other consumers. The time is right, we believe, to renew long-discarded efforts to systematically address humanistic elements of the evidence-based delivery of ABA treatment in autism. The framework of BA offers an opportunity to expand both the repertoires of effective practitioners and the future dimensions of ABA.
